# Mitochondrial Genomic Analysis of Late Onset Alzheimer’s Disease Reveals Protective Haplogroups H6A1A/H6A1B: The Cache County Study on Memory in Aging

**DOI:** 10.1371/journal.pone.0045134

**Published:** 2012-09-17

**Authors:** Perry G. Ridge, Taylor J. Maxwell, Christopher D. Corcoran, Maria C. Norton, JoAnn T. Tschanz, Elizabeth O’Brien, Richard A. Kerber, Richard M. Cawthon, Ronald G. Munger, John S. K. Kauwe

**Affiliations:** 1 Department of Biology, Brigham Young University, Provo, Utah, United States of America; 2 ARUP Institute for Clinical and Experimental Pathology, Salt Lake City, Utah, United States of America; 3 Human Genetics Center, University of Texas School of Public Health, Houston, Texas, United States of America; 4 Department of Mathematics and Statistics, Utah State University, Logan, Utah, United States of America; 5 Center for Epidemiologic Studies, Utah State University, Logan, Utah, United States of America; 6 Department of Family Consumer and Human Development, Utah State University, Logan, Utah, United States of America; 7 Department of Psychology, Utah State University, Logan, Utah, United States of America; 8 Department of Epidemiology and Population Health, University of Louisville, Louisville, Kentucky, United States of America; 9 Department of Human Genetics, University of Utah, Salt Lake City, Utah, United States of America; 10 Department of Nutrition, Dietetics, and Food Sciences, Utah State University, Logan, Utah, United States of America; Pasteur Institute of Lille, France

## Abstract

**Background:**

Alzheimer’s disease (AD) is the most common cause of dementia and AD risk clusters within families. Part of the familial aggregation of AD is accounted for by excess maternal vs. paternal inheritance, a pattern consistent with mitochondrial inheritance. The role of specific mitochondrial DNA (mtDNA) variants and haplogroups in AD risk is uncertain.

**Methodology/Principal Findings:**

We determined the complete mitochondrial genome sequence of 1007 participants in the Cache County Study on Memory in Aging, a population-based prospective cohort study of dementia in northern Utah. AD diagnoses were made with a multi-stage protocol that included clinical examination and review by a panel of clinical experts. We used TreeScanning, a statistically robust approach based on haplotype networks, to analyze the mtDNA sequence data. Participants with major mitochondrial haplotypes H6A1A and H6A1B showed a reduced risk of AD (p = 0.017, corrected for multiple comparisons). The protective haplotypes were defined by three variants: m.3915G>A, m.4727A>G, and m.9380G>A. These three variants characterize two different major haplogroups. Together m.4727A>G and m.9380G>A define H6A1, and it has been suggested m.3915G>A defines H6A. Additional variants differentiate H6A1A and H6A1B; however, none of these variants had a significant relationship with AD case-control status.

**Conclusions/Significance:**

Our findings provide evidence of a reduced risk of AD for individuals with mtDNA haplotypes H6A1A and H6A1B. These findings are the results of the largest study to date with complete mtDNA genome sequence data, yet the functional significance of the associated haplotypes remains unknown and replication in others studies is necessary.

## Introduction

Alzheimer’s disease (AD) is a complex disorder and is the most common form of dementia [1]. After age, family history is the single greatest risk factor for AD. AD can be classified into early and late onset forms. Mutations in three genes: PSEN1/2 and APP are known to cause early onset AD in an autosomal dominant manner [2], [3]. The majority of AD cases, however, are late onset (LOAD) and the APOE e4 allele is the strongest known genetic risk factor. Many additional genetic polymorphisms have been identified, though with substantially lower risk estimates [1], [4], [5], [6], [7], [8], [9], [10]. LOAD appears to be inherited and/or sporadic and there is evidence of a maternal inheritance pattern [11]. Current estimates suggest that more than 20% of inherited LOAD cases are maternally inherited [12].

Analyses of families with inherited LOAD have repeatedly reported a greater incidence of AD in children with affected mothers than with affected fathers. Among individuals affected with AD who have one affected parent, the mother is 1.8 to 3.8 times more likely to be affected than the father [13], [14], [15], [16]. When extended to families with multiple affected siblings and a single affected parent, the ratio of affected mothers to fathers increased to 9∶1 [13]. While no biological mechanisms for maternal inheritance were demonstrated, results of these studies, observed in multiple datasets, strongly suggest maternal inheritance.

Imaging studies provide additional evidence of maternal inheritance of AD. These studies have identified decreased glucose metabolism and atrophy in brain regions affected in AD. Similar to the studies cited above, individuals with a paternal, maternal, or no family history of AD were compared. First, progressive gray matter atrophy was only observed in people with a maternal family history of AD. These same individuals had greater atrophy in the precuneus and parahippocampal gyrus (regions known to be affected in AD) than those with a paternal or no family history of AD [17]. Next, other studies compared reductions in glucose metabolism in the brain for each of the three groups listed above. Subjects with a paternal family history of LOAD had decreased glucose metabolism similar to those with no family history; however, individuals with a maternal family history of LOAD had significantly decreased glucose metabolism compared to the other groups [18], [19]. Additionally, similar to the atrophy studies, lowered glucose metabolism was concentrated in the same brain regions known to have impaired glucose metabolism in AD (posterior cingulate cortex/precuneus, parieto-temporal, and medial temporal cortices) [18], [19]. The increased incidence of AD or risk for AD-related phenotypes among individuals with a maternal family history of AD, compared to people with no family history or a paternal family history of AD, strongly support a maternal inheritance pattern for LOAD.

Maternal inheritance occurs by several mechanisms including disease susceptibility genes on the X-chromosome, maternal specific genetic imprinting, or by mitochondrial genetic effects. We investigated the role of mitochondrial sequence variants in maternal transmission of LOAD. Mitochondrial malfunction is a plausible explanation for a number of AD phenotypes, including the decreased glucose metabolism in specific brain regions discussed above. Numerous mitochondrial modifications in patients with AD have been reported; these include morphological changes [20], [21], alterations in the enzymes of the electron transport chain, including cytochrome c oxidase [22], [23], changes in the mitochondrial proteome [24], and reduced numbers of mitochondria [22]. Beta-amyloid plaques aggregate within mitochondria [25], [26] and it has been hypothesized that changes in mitochondrial function facilitate Aβ deposition and tau phosphorylation [27]. These observations have led investigators to ask whether mitochondrial dysfunction is a cause or effect of plaque aggregation.

The mitochondrial cascade hypothesis [28] posits that a decline in mitochondrial number and function is a cause of neurodegeneration. Briefly, it is known that mitochondrial function declines with age and in conjunction with certain morphological changes [20], [21]. As mitochondrial function declines with age, hypothesized consequences are increased tau phosphorylation and beta-amyloid amyloidosis in brain tissue. In contrast, in familial forms of AD, Aβ aggregation and tau phosphorylation are hypothesized to occur before mitochondrial malfunction and lead to the mitochondrial dysmorphology and dysfunction characteristic of AD [21], [28], [29]. Other evidence suggests that Aβ aggregation directly causes mitochondrial malfunction [30], [31] or that Aβ and tau interact to increase oxidative stress and impede mitochondrial function [32].

Mitochondrial malfunction can be caused by numerous factors, one of which is inherited sequence variation in the mitochondrial genome. To date, many studies have been published analyzing the association between mitochondrial haplogroups or specific mitochondrial sequence variants, and AD. The results have been mixed, confusing, and at times contradictory. The majority of studies have not identified any associations [33], [34], [35], [36], [37], [38], but some have reported significant associations. Haplogroups H and U (or sub-haplogroups of H and U) have been associated with both increased and decreased risk of AD [39], [40], [41], [42], [43] and different effects for men and women. The UK and HV clusters, as well as haplogroups J, G2A, B4C1, and N9B1, have been associated with increased risk for AD [44], [45], [46], [47], [48], while haplogroups K and T are thought to be protective [40], [48]. No consensus has been reached and no previous studies have reported on large population-based samples with complete mtDNA genome sequence data. Here we present the largest analysis to date of mitochondrial haplotypes and associated risks for LOAD based on fully sequenced mitochondrial genomes.

## Materials and Methods

### Ethics Statement

All study procedures were approved by the Institutional Review Boards of Brigham Young University, Utah State University, Duke University, and Johns Hopkins University. Written consent was obtained for each individual. To verify a subject’s capacity to consent, subjects attempted the Modified Mini-Mental State Exam (3MS). If there was an indication of poor cognitive ability as determined by poor performance on the entire test (scoring below a designated total of 60 points), poor performance on temporal or spatial orientation, or clear difficulty in understanding the nature of the interview, the visit was discontinued and informed consent was obtained from a responsible caregiver–often the next-of-kin. We re-consented subjects/caregivers at each study visit and procedure.

### Sample Acquisition and Sequencing

The Cache County Study on Memory in Aging was initiated in 1994 to investigate the occurrence of dementia and associations with APOE genotype, environmental exposures, and cognitive function. A cohort comprised of 5,092 Cache County, Utah, residents was established and followed continually for 12 years. The cohort represents a 90% sample of all residents of Cache County who were aged 65 and older in 1994. Over the 12-year follow-up period, data were collected in four (triennial) waves. Data collected in each wave included basic demographic information, family and medical histories, and a multistage dementia assessment screen; in addition, a more detailed clinical assessment was done for: a) those who screened positive for AD according to the multistage screening protocol for dementia; or b) were randomly selected, according to age, gender, and APOE genotype, to complete all stages of assessment [49]. Diagnoses of dementia were based on expert clinical assessments, standard MRI, and laboratory studies. Diagnoses of Probable or Possible AD were based on NINCDS-ADRDA criteria [50]. Those without dementia were diagnosed as “non cases” following a clinical assessment (if the individual was a designated subsample member), or if lacking a clinical assessment, screened negative at each stage of dementia screening and evaluation. Any participant who screened positive at any screening stage, but failed to complete the next stage of screening was removed from the analyses. For individuals without clear non-demented or Alzheimer’s disease diagnosis case-control status was set to missing or unknown.

The Utah population is the source of most of the Centre d’Etude du Polymorphisme Humain (CEPH) families. The CEPH families have been used in countless genetic studies to represent Caucasians worldwide, or for example, to represent the “European” sample assayed by the HapMap project [51]. The genetic structure of the Utah population is broadly representative of other U.S. populations of northern European ancestry characterized by very little inbreeding. Utah’s founding pioneer group was relatively large, migrated from many different points of European origin, and overall, was largely unrelated [52], [53], [54].

Cache County study participants were linked to the Utah Population Data Base and each participant was assigned to a unique matrilineage with a common maternal founder. The matrilineages were rank ordered by size (total number of known members) and a single Cache County Study participant was randomly selected from each of the Cache County matrilineages. The number of matrilineages sequenced was limited by the available funding; we started with the largest matrilineage and worked down the list. The participants selected for mtDNA sequencing were selected independent of their cognitive or dementia status. 274 matrilineages were represented by this dataset. As a result, the sequenced mitochondrial genomes also represent as many different major mitochondrial haplogroups and clusters as possible (Table 1). Selection was made blind to case-control status. 287 samples were sent to Family Tree DNA (www.familytreedna.com) for Sanger sequencing of the mitochondrial genomes. Family Tree DNA applied QC criteria, and 285 of 287 passed QC. Family Tree DNA also reported which of the known mitochondrial haplogroups or clusters each of these individuals belonged to. We then identified each of the unique haplotypes, and their frequencies, in our dataset. In many instances several of the unique haplotypes in our dataset all belonged to the same major mitochondrial haplogroup/cluster (or sub-haplogroup). In this manuscript major mitochondrial haplogroups/clusters and sub-haplogroups refer to known mitochondrial haplotypes/clusters (i.e. H, H6A1A, HV, J, etc.) and haplotypes refer to the distinct mitochondrial haplotypes identified in our dataset. Among the 285 individuals whose mitochondrial genomes were sequenced were eight cases, 197 controls, and 80 individuals with missing AD case-control status.

**Table 1:** Distribution of major mtDNA haplogroups/clusters.

Major Haplogroup	Number	Cases	Controls	Missing^1^	Ethnicities [55], [68]
H	424	55	264	105	European
U	147	12	88	47	European
T	121	7	74	40	European
J	99	12	60	27	European
K	95	8	65	22	European
V	34	1	24	9	European
I	21	2	10	9	European
W	20	1	15	4	European
HV	18	2	14	2	European
X	8	0	8	0	European
C	5	1	2	2	Asian
L	4	0	2	2	African
Missing^2^	11	0	6	5	

Here we report the number of individuals belonging to each of the major haplogroups represented in our dataset along with case-control status.

1 Missing case-control status.

2 Missing major haplogroup.

### Analyses

Following sequencing we were provided with a list of mtDNA sequence variants for each of the patient samples. Whole mtDNA sequences were generated for each person using Java programs to map variant sites back to a reference mtDNA sequence (NC_012920), and MitoMap annotations were used for describing gene coordinates [55]. A number of sites were heteroplasmic, and for these we considered only the most common of the two possible alleles. Our dataset contained a total of 285 full mitochondrial sequences of high quality. Using extended pedigree information from the Utah Population Database [56], we identified individuals sharing maternal lineage membership with those who were directly genotyped. Based on these pedigree (matrilineal) relationships, we were able to impute an additional 722 genome sequences for a total of 1007 full mitochondrial genome sequences.

ClustalW [57] was used to align the mitochondrial genomes. Using the 285 genotyped individuals, we inferred a haplotype network using TCS [58]. Genotype-phenotype associations were evaluated using an evolution-based method known as TreeScanning [59], [60] that makes use of haplotype networks. Such networks provide an *a priori* framework from which to pool haplotypes based on common descent with the assumption that a mutation causing a phenotypic effect is embedded within the same historical structure represented by the haplotype network [61]. Practically, TreeScanning uses each branch of the haplotype network to define a set of bi-allelic partitions. For each branch, haplotypes are pooled together into one of two allelic classes depending on which side of the branch they occur. Individual genotypes are then determined by which “alleles” they have. Being haploid, there are only two genotypes possible (i.e. no heterozygote class). This is done for each branch in the tree, resulting in a set of correlated tests. Significance levels were corrected for using a permutation analog of the step-down, stepwise Bonferroni method [62], which takes into account the correlations among tests, and allows for more than one significant test. A second round of TreeScanning conditional on a significant branch from the first round is used to determine if other significant branches represent the same or different effects, or possibly additional associations masked by effects in the previous round of TreeScanning. The original TreeScanning method [59] dealt with univariate continuous data.

For our case-control data we used logistic regression [63], [64], [65] with age, gender, familial risk, and APOE genotype as covariates. Familial risk scores were computed for each study subject as the weighted sum of biological relatives who were diagnosed with AD divided by the weighted sum of biological relatives who were members of the at-risk cohort, using the coefficient of relationship (twice the kinship coefficient) as a weighting function [66]. Each analysis was performed with 10,000 permutations. Only tests with at least two relevant genotypic classes, each containing five or more individuals, were tested. This was done to exclude tests with little power to detect associations and to increase statistical power overall by reducing the total number of tests. Significance was inferred if the multiple-test-corrected p-value was less than 0.05.

## Results

### Haplotype Network and mtDNA Variation

We observed 249 different haplotypes in our sample of 1007 full mitochondrial genomes (mtDNA). The majority of haplotypes (152 of 249) were observed in three or fewer individuals and the two most frequently observed haplotypes consisted of 39 and 32 individuals, respectively. The most frequently observed haplotype (39 individuals) was the root of our haplotype network. Our network (Figure S1) contained one unresolved loop. This loop was left in the network and the ambiguity factored into all subsequent analyses.

We identified 899 single nucleotide variants (SNVs), 26 insertions, and 20 deletions in our dataset. The most frequently observed SNVs occurred in 281 genomes (m.263A>G, m.8860A>G, and m.15326A>G), and three more SNVs were observed in 280 genomes (m.750A>G, m.1438A>G, and m.4769A>G). Compared to the reference sequence (NC_012920), the average number of variants per individuals was 25.3; one individual had the highest number of variants (52), and one individual the fewest (2).

Lastly, we analyzed the distribution of major mitochondrial haplogroups within our dataset (Table 1, Table S1). Individuals in our dataset corresponded by haplotype to 102 major mitochondrial haplogroups/clusters (or sub-haplogroups), and as expected, the majority (987 of 1007) belonged to European based major haplogroups.

### mtDNA Variation Associated with Alzheimer’s Disease Risk

**Figure 1:**
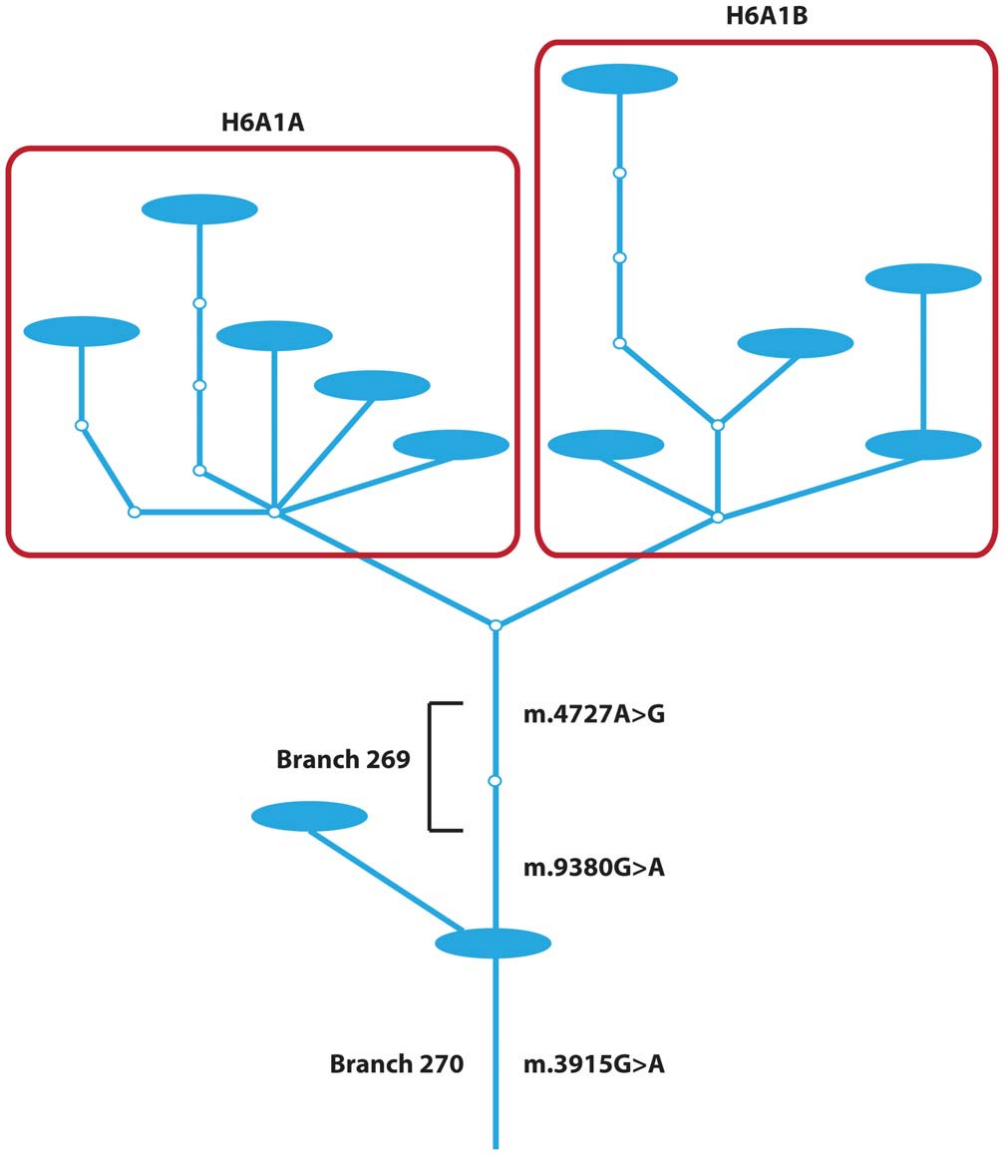
Branches 269 and 270. Here we show branches 269 and 270 from our network (Figure S1). Branch 270 consists of a single segment and 269 two segments, where each segment corresponds to a single variant. The two branches are labeled along with the variants corresponding to these branches. Together these two branches represent a single significant association. The other branches/variants are not labeled since they were not significantly associated with Alzheimer’s disease case-control status. The nodes corresponding to major mitochondrial haplogroups H6A1A and H6A1B are enclosed by red rectangles and are labeled. Finally, observed haplotypes within our dataset are represented by the blue ellipses.

In a haplotype network each segment of a branch (and a branch can have multiple segments) corresponds to a specific sequence feature, and each individual appearing below a segment of the network shares the particular feature that corresponds to the branch segment. In our study, two different branches were significantly associated with AD (see Tables 2 and 3) in the first round of TreeScanning. As shown in Figure 1, the clade defined by branch 269 is nested within the clade defined by branch 270; as a result, these two highly correlated branches represent a single significant association. The clade defined by branch 270 consists of 38 individuals and 11 distinct haplotypes. These individuals were 73.85 (standard deviation 7.55) years old on average, and 13 of 38 were male. Among the 38 individuals of branch 270 there were seven groups of siblings, one group of three siblings, and six sets of sibling pairs; 23 individuals had no siblings in the dataset. We classified each of the individuals into major mitochondrial haplogroups. Of the 38 branch 270 individuals, one belonged to major haplogroup H, 12 belonged to H6A1A, and 25 belonged to H6A1B. None of the 38 individuals of branch 270 tested positive for AD, an absence of cases that makes this group of individuals, branch 270, significantly different in comparison to all other branches of the haplotype network (corrected p-value 0.016).

**Table 2:** Demographic characteristics of selected participants in the Cache County Study of Memory in Aging.

	Number	Age (St dev)	Gender (male/female)	Cases/Controls/Missing
Total Individuals	1007	75.64 (7.50)	442/565	101/632/274
Branch 270	44	74.56 (8.11)	14/30	0/38/6
Branch 269	42	74.62 (8.29)	13/29	0/37/5

Here we report demographic information for total individuals and for individuals in each of two contrasts in our dataset, including the number of cases, controls, and those with no phenotype. Ages were based on collection date of blood.

**Table 3:** Information about the significant contrasts.

Contrast	p-values no family risk		p-values with family risk		Defining variant(s)
	nominal	corrected	nominal	corrected	
Branch 270	1.0E-04	0.016	3.0E-04	0.017	m.3915G>A (p.G213G)
Branch 269	1.0E-04	0.018	3.0E-04	0.021	m.4727A>G (p.M86M)m.9380G>A (p.W58W)

Reported here are the two contrasts. Two sets of p-values are reported. First, uncorrected and corrected p-values without the family risk for AD covariate, and p-values after adding the family risk covariate. These two contrasts are not independent and effectively represent the same single significant association.

Since the clades defined by the two branches (269 and 270) are statistically indistinguishable, so too are the three variants (Figure 1) corresponding to these two branches. The sequence variant, m.3915G>A (p.G213G), that defines branch 270, is in the NADH dehydrogenase I gene (ND1) and has an estimated population frequency of ∼1% [67]. This branch 270 variant (m.3915G>A) has been observed in three of the major mitochondrial haplogroups: B (Asian), H (European), and L3 (African) [68]. Branch 269 is two segments long and thus is defined by two variants: m.4727A>G (p.M86M) is located in the NADH dehydrogenase 2 gene (ND2); and m.9380G>A (p.W58W) is located in the cytochrome c oxidase III gene (COX3). Both are synonymous polymorphisms that have been observed previously [68]. m.9380G>A differentiates major haplogroup H6A1 (from H6A), and m.4727A>G is also specific to H6A1 [69].

Our data contained seven sib groups (6 pairs and a trio) among the individuals contained in branches 269 and 270 as well as more distant relationships among some individuals without sibs. In order to verify that family risk for AD was not the basis of the observed associations, we added an additional covariate, family risk for AD (see Methods), to our model and reran our analyses. Even after controlling for family risk, these two contrasts remained significant (corrected p-value = 0.0174 and 0.0213 for 270 and 269, respectively).

## Discussion

Here we present evidence for decreased risk of AD for complete mitochondrial haplotypes categorized within haplogroups H6A1A and H6A1B. Results from analyses, whether or not controlling for relatedness, were consistent; therefore our approach is robust when considering the familial relationships within the sample. The protective haplotypes were defined by three variants: m.3915G>A, m.4727A>G, and m.9380G>A. These three variants characterize two different major haplogroups. Together m.4727A>G and m.9380G>A define H6A1, and it has been suggested m.3915G>A defines H6A [69], although that is not certain as it has been observed outside major haplogroup H6A [68]. Additional variants differentiate H6A1A and H6A1B; however, none of these variants had a significant relationship with AD case-control status.

We know of no known functional explanation for these three variants or two major haplogroups that provide protection against AD. Each of the three variants is synonymous, and none of the three are located close to the 5′ end of a gene. All three, however, are located in genes important for oxidative phosphorylation. ND1 and ND2 are components of NADH dehydrogenase, or Complex I, the first and largest enzyme in the electron transport chain [70]. Lin and colleagues reported mutations in codon 331 of ND2 in the brains of AD patients; however, this hasn’t been replicated and the sample size was very small (19 cases and 11 controls) [71]. The observed variant in ND2 in our dataset was in codon 86 of ND2. The third variant was located in COX3, a critical transmembrane component of cytochrome c oxidase (complex IV), the final enzyme in the electron transport chain [72]. Given the evidence of changes in the electron transport chain in AD patients [22], [23], variants affecting the efficiency of respiration could plausibly contribute to or protect against AD.

There is no consensus on the effect of specific mitochondrial haplogroups for risk of AD and there have been mixed results for haplogroup H (and cluster HV). In one study haplogroup H was used as the reference group (since it was the most common in the dataset), and males with haplogroup U had increased risk for AD, while haplogroup U females had decreased risk compared to haplogroup H [39]. These data are consistent with a possible protective effect for haplogroup H in males, although the study was based on only 10 SNPs and consequently did not provide more resolution. Another study found significant enrichment for AD cases in haplogroup H, but only a portion of the D-loop was sequenced and the sample size was small [41]. In yet another study of D-loop sequences and specific coding region markers, H5 individuals were enriched for AD cases compared to other haplogroups [42]. Maruszak et al [45] found an association for the HV cluster with AD, but concluded that further analysis is necessary in order to understand the precise relationship of different haplotypes with AD. Lastly, the GERAD Consortium, in a very large study (∼3000 cases and ∼1000 age matched controls), tested 138 mtDNA markers, but found no significant associations with AD [38]. While the GERAD study had a very large sample size it had insufficient markers to test the groups we tested. One possible cause of the ambiguity in previous research is that in most cases only major mitochondrial haplogroups and/or clusters were analyzed; however, within each major haplogroup are many different haplotypes. By using the TreeScanning method we were able to overcome this challenge and identify which sub-haplogroups specifically provided protection against AD.

We have presented the results of association analyses using full mitochondrial sequences. Use of this complete mitochondrial genomic data allowed more precise identification of sequence variants for use in association studies. Furthermore, we were able to map these individuals back to two previously defined haplogroups: H6A1A and H6A1B. Our findings provide evidence of a reduced risk of AD for individuals of the two haplogroups defined by the three reported variants. This is the largest study to date using full mitochondrial genome sequences to look for association with LOAD. These data lack replication from an independent study and warrant validation in other datasets with a more diverse sampling of major haplogroups.

## Supporting Information

Figure S1 **Haplotype network.** Here we show our entire haplotype network based on all the full mitochondrial genome sequences in our dataset, and have enclosed the clades corresponding to branches 269 (red square) and 270 (red square and circle).
(PDF)

Table S1 **Major mitochondrial haplogroups, clusters, and sub-haplogroups.** Here we have listed the haplogroup membership of all 1007 individuals in our dataset. The numbers inside the parentheses are the number of individuals with a major mitochondrial sub-haplogroup and the first column shows the membership in each of the represented major mitochondrial haplogroups in our dataset.
(XLSX)
