# Supplementary figures and images for: Mitochondrial Genomic Analysis of Late Onset Alzheimer’s Disease Reveals Protective Haplogroups H6A1A/H6A1B: The Cache County Study on Memory in Aging
Source: PLoS One. 2012 Sep 17;7(9):e45134. doi: 10.1371/journal.pone.0045134 (PMC3444479; doi:10.1371/journal.pone.0045134)

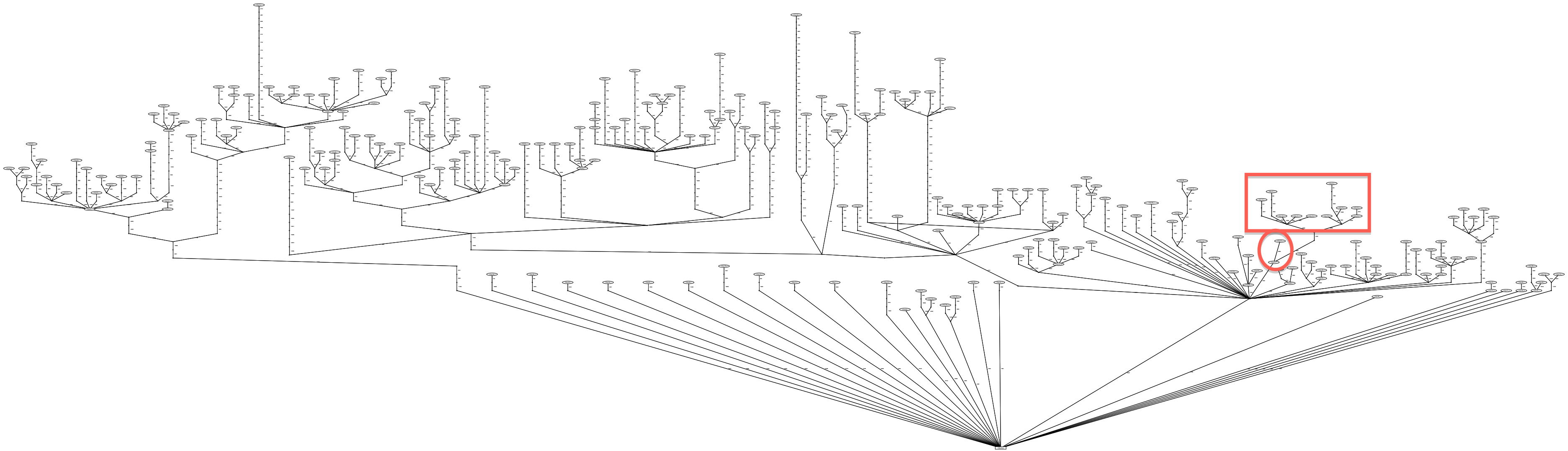

Supplement: Figure S1 — Haplotype network. Here we show our entire haplotype network based on all the full mitochondrial genome sequences in our dataset, and have enclosed the clades corresponding to branches 269 (red square) and 270 (red square and circle). (PDF) [file pone.0045134.s001.pdf]
